# Wavelength Optimization for Quantitative Spectral Imaging of Breast Tumor Margins

**DOI:** 10.1371/journal.pone.0061767

**Published:** 2013-04-16

**Authors:** Justin Y. Lo, J. Quincy Brown, Sulochana Dhar, Bing Yu, Gregory M. Palmer, Nan M. Jokerst, Nirmala Ramanujam

**Affiliations:** 1 Department of Biomedical Engineering, Duke University, Durham, North Carolina, United States of America; 2 Department of Electrical and Computer Engineering, Duke University, Durham, North Carolina, United States of America; 3 Department of Radiation Oncology, Duke University Medical Center, Durham, North Carolina, United States of America; National Cancer Center, Japan

## Abstract

A wavelength selection method that combines an inverse Monte Carlo model of reflectance and a genetic algorithm for global optimization was developed for the application of spectral imaging of breast tumor margins. The selection of wavelengths impacts system design in cost, size, and accuracy of tissue quantitation. The minimum number of wavelengths required for the accurate quantitation of tissue optical properties is 8, with diminishing gains for additional wavelengths. The resulting wavelength choices for the specific probe geometry used for the breast tumor margin spectral imaging application were tested in an independent pathology-confirmed *ex vivo* breast tissue data set and in tissue-mimicking phantoms. In breast tissue, the optical endpoints (hemoglobin, β-carotene, and scattering) that provide the contrast between normal and malignant tissue specimens are extracted with the optimized 8-wavelength set with <9% error compared to the full spectrum (450–600 nm). A multi-absorber liquid phantom study was also performed to show the improved extraction accuracy with optimization and without optimization. This technique for selecting wavelengths can be used for designing spectral imaging systems for other clinical applications.

## Introduction

A wavelength optimization strategy is developed to improve the design of a novel spectral imaging probe array [1] for quantitative assessment of breast tissue margins during partial mastectomy surgery, a common treatment for early stage breast cancer [2], [3]. This generalized method is based on a search heuristic known as a genetic algorithm that mimics the process of natural evolution and identifies reduced wavelength sets that maintain tissue optical contrast when compared to the broadband data. It requires a technique for measuring or simulating spectral data with known optical contrast and a metric for data extraction quality. Diffuse reflectance spectroscopy in the visible range can be used to non-destructively measure tissue optical properties. The propagation of multiply scattered photons is sensitive to the absorption by biological molecules and can ultimately provide contrast between adipose tissue content (β-carotene absorption), vascularity (hemoglobin absorption) and scattering (fibroglandular content). During partial mastectomy, the surgeon strives to excise the entire tumor with a surrounding rim (or “margin”) of normal tissue while preserving as much normal tissue as possible in the breast. Ultimately, the complete removal of the breast tumor is vital to reducing the chance of tumor recurrence [4]. A previously developed spectral imaging system for breast tumors consists of a broadband illumination source, an 8-channel fiber optic conduit to direct light to and from the tissue, and an imaging spectrograph and cooled CCD for detection. Each placement of the imaging probe allowed for diffuse reflectance spectra (450–600 nm) to be measured from up to 8 sites on the margin. Multiple placements of the probe allowed for mapping the entire margin surface. Optical properties of the specimens were extracted to create tissue composition maps of total hemoglobin concentration, β-carotene concentration, and tissue scattering using a fast, scalable Monte Carlo model of reflectance previously developed by our group [5], [6]. Pathologically-confirmed positive margins, showed significantly lower β-carotene/scattering ratios compared to negative margins. This finding reflects a decrease in fat content and an increase in fibroglandular content associated with margin positivity. The sensitivity and specificity of the system for determining margin status was 79% and 67%, respectively [7].

The clinical adaptability of this technology will be impacted by its size, cost, and the time needed to diagnostically map tumor margins. This motivated the design of a compact and cost-effective device based on the utilization of a few discrete wavelengths for illumination to replace a broadband source and monochromator in the original system and inexpensive photodiode arrays for detection in lieu of a spectrograph and CCD camera [8]–[10]. Optimized selection of wavelengths and bandpass filters was important to minimize complexity and acquisition time, while maintaining comparable sensitivity to the relevant sources of optical contrast in the breast. Several groups have reported on the optimization of wavelength combinations for specific clinical applications. Using a matrix decomposition of basis spectra and simulation of tissue data, Mazhar et al. optimized wavelength pairs to measure hemodynamic changes in the near-infrared range for breast imaging applications with diffuse optical tomography [11]. By solving a linear equation based on a modified Beer-Lambert Law, Umeyama and Yamada accounted for cross-talk of measured NIR chromophores in wavelength combinations for studying the brain [12]. Ferreira et al. presented a device fabrication driven strategy for the spectroscopic imaging of esophageal tissue, featuring 16 discrete wavelengths in the 350–750 nm range [13]. The selection of wavelengths was constrained by the filter fabrication process, i.e. materials, number of layers, FWHM, etc. Phelps et al. developed a ratiometric method that involves the selection of wavelength pairs that are independent of tissue scattering to rapidly estimate total hemoglobin concentration in the UV-visible range [14]. These previous studies show the importance of optimizing wavelength selection for various clinical applications. Although diffuse reflectance and elastic scattering spectroscopy [15] have increasingly been applied to breast tumor margin assessment, few have optimized system production for clinical translation. In this manuscript, a strategy is presented for optimizing wavelength selection for a cost-effective design of spectral devices for tissue margin assessment.

## Methods

**Figure 1:**
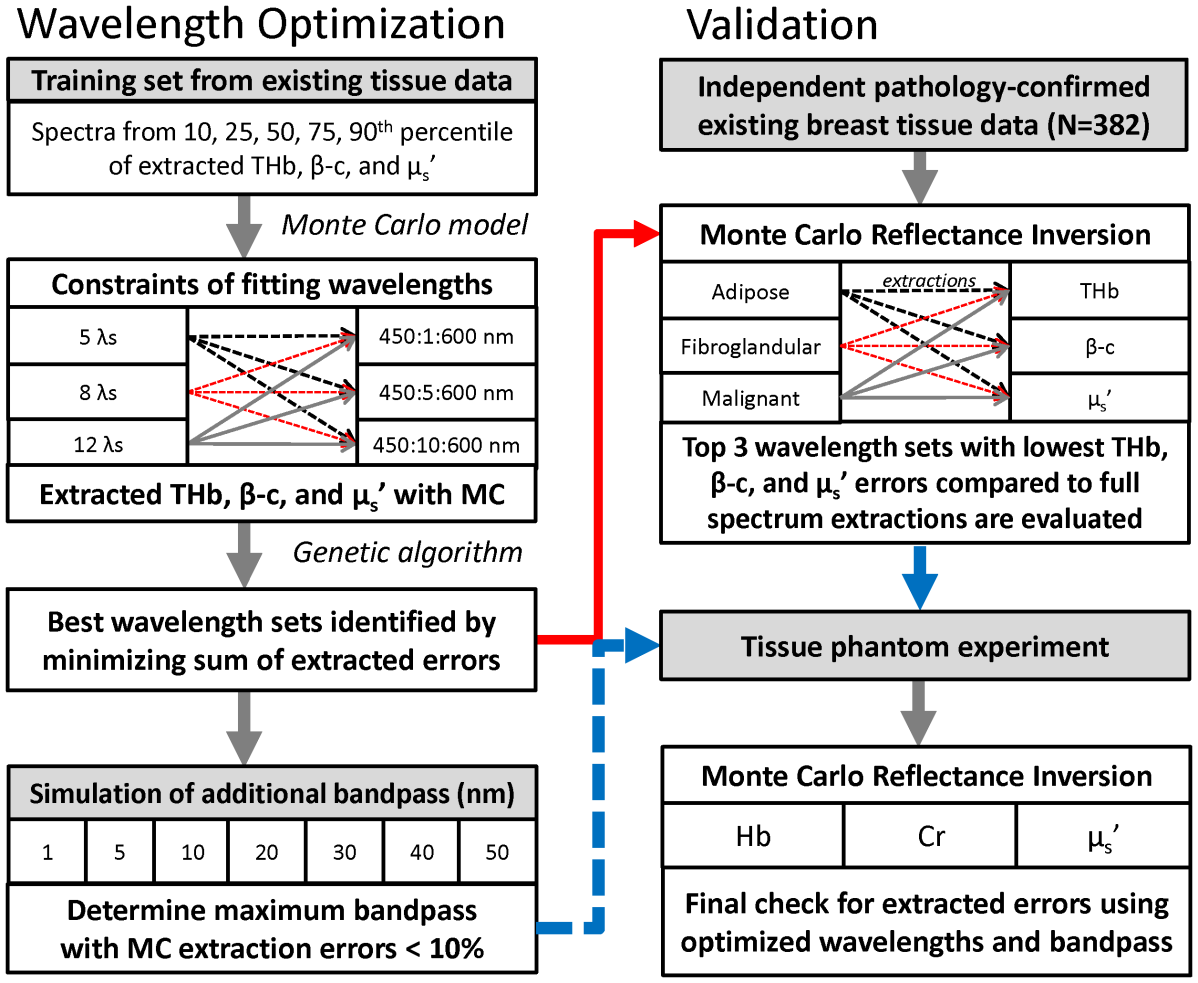
General flow chart of wavelength selection method. General flow chart illustrating the process for selecting and testing optimal wavelength sets and spectral bandpass in clinical data obtained from breast tumor specimens and in tissue phantoms.

Figure 1 provides a broad overview of the steps taken to determine and test the best wavelength sets and spectral bandpass. First, existing *ex vivo* breast tissue reflectance data was used as a training set for the optimization algorithm. A fast, scalable Monte Carlo reflectance model [5] was used to extract the tissue optical properties from the training set using various constraints, such as the total number of wavelengths, the range of wavelengths, and the increment of each wavelength from which to select. Combined with a genetic algorithm for global optimization, the best wavelength sets are identified by minimizing the sum of tissue property extraction errors from the reflectance spectra. The selected wavelengths are then validated with an independent pathology-confirmed *ex vivo* breast tissue data set. A tissue-mimicking phantom experiment was also performed as part of the wavelength selection validation. The text in these subsequent sections describes the methods in greater detail.

### 1. Wavelength Optimization

#### 1.1 Diffuse reflectance spectra from ex vivo breast tissue specimens

**Figure 2:**
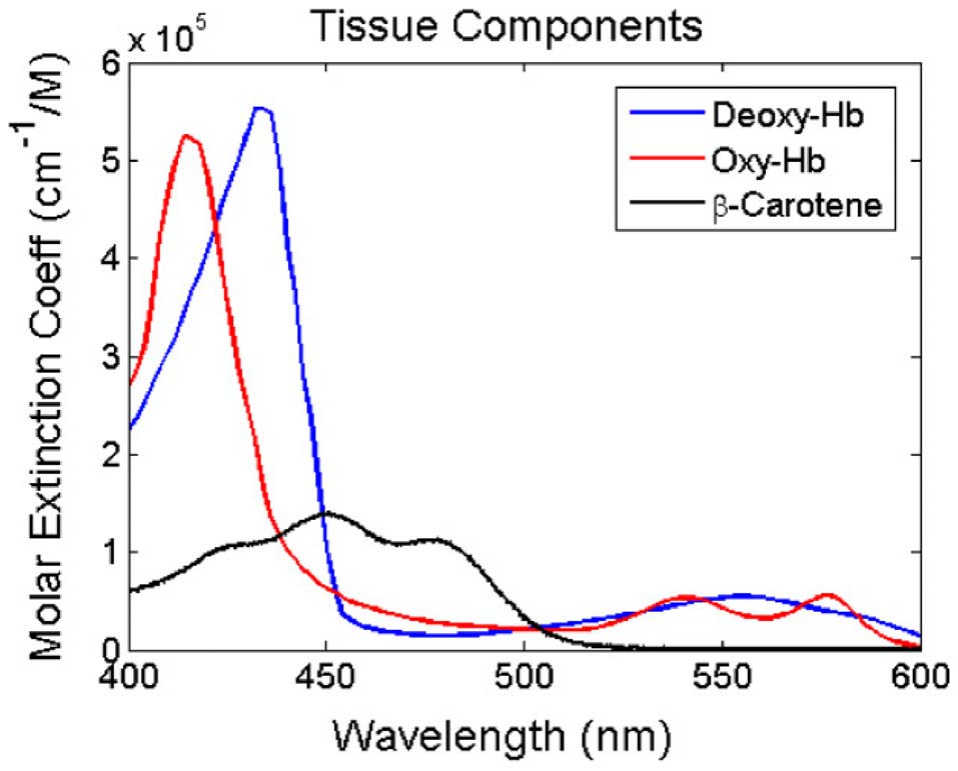
Dominant absorbers of breast tissue in the UV-visible spectrum. Molar extinction coefficient of oxy- and deoxy- hemoglobin and β-carotene in the 400–600 nm range.

The dominant absorbers in the visible spectrum in breast tissue are oxy- and deoxy-hemoglobin and β-carotene. The absorption spectra of these breast tissue components are shown in Figure 2. Previous studies have shown that β-carotene and tissue scattering are significant parameters that can be used to differentiate between malignant and benign breast tissues [2], [16], [17]. To select the minimum set of wavelengths in the visible spectral range that are sensitive to these key tissue constituents, an existing data set of 4953 diffuse reflectance spectra measured from an *ex vivo* clinical study at Duke University Medical Center approved by the Duke University Institutional Review Board (protocol #00017428) involving partial mastectomy procedures on 100 patients was used as a training set for the wavelength optimization [7], [17]. The diagnosis for the 101 margins were as follows: 44 negative (>2 mm normal tissue), 35 close (<2 mm normal tissue), and 22 positive. In addition, routine histopathology was performed on a subset of these measurements, and the study pathology classified 6–10 randomly selected measurement locations (or “sites” on the margins). A total of 320 normal adipose sites, 24 normal fibroglandular sites, and 38 malignant sites were used for testing the optimized wavelength sets.

Total hemoglobin [THb], β-carotene [βc], and reduced scattering coefficients <µ_s_’> for each of the 4953 diffuse reflectance spectra were extracted using a previously developed inverse Monte Carlo model of reflectance [5], [6]. Reflectance spectra of samples at the 10^th^, 25^th^, 50^th^, 75^th^, and 90^th^ percentile of the empirical cumulative distribution functions (cdf) of [THb], [βc], and average <µ_s_’> were chosen resulting in a total of 15 reflectance spectra in the training set. This method ensured that the data are sampled evenly over the distributions rather than the parameter value ranges, which could result in oversampling of samples at the periphery of the distributions.

Table 1 lists the extracted breast tissue properties for each of the 15 selected reflectance spectra for the wavelength optimization training set. Samples 1–5, 6–10, and 11–15 represent the 10^th^ through 90^th^ percentiles of [THb], [βc], and average <µ_s_’>, respectively. The objective in selecting reflectance spectra based on the cdfs extracted from an extensively large data set of previously measured *ex vivo* breast tumor margins was to cover a wide range of [THb] (10.7–97.9 µM), [βc] (7.0–37.6 µM), and average <µ_s_’> over 450–600 nm (3.7–11.9 cm^−1^). Thus, from the 15 reflectance spectra, each with 3 extracted parameters to compare, there are 45 individual parameters to compare between the reduced wavelength spectrum extractions and the full spectrum extractions.

**Table 1:** Extracted *ex vivo* breast tissue properties used for training set.

Sample	[THb] (µM)	[β-carotene] (µM)	<µ_s_’>_450–600_ (cm^−1^)
1	10.7	7.5	4.1
2	18.1	21.7	5.3
3	32.7	18.6	11.5
4	59.3	21.0	8.0
5	97.9	16.6	4.4
6	55.7	7.0	3.9
7	49.6	11.3	9.2
8	32.8	17.5	6.3
9	73.1	26.0	8.8
10	95.9	37.6	8.4
11	24.2	13.6	3.7
12	22.3	29.3	4.8
13	40.3	31.3	6.5
14	91.1	15.8	8.9
15	11.5	30.8	11.9

#### 1.2 Combined monte carlo reflectance model and genetic algorithm to select center wavelengths

**Figure 3:**
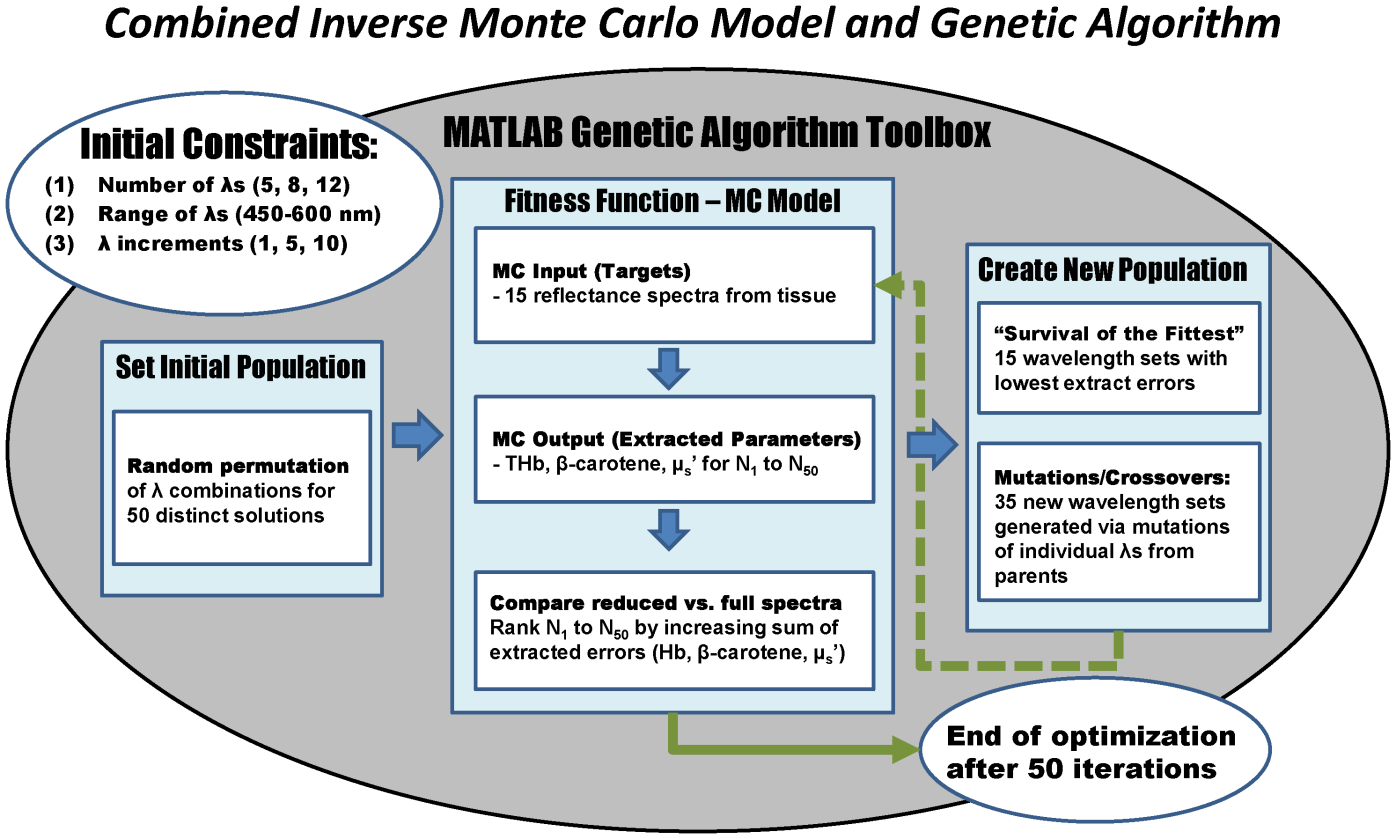
Diagram of combined Monte Carlo reflectance model and genetic algorithm. Diagram detailing the steps of selecting wavelengths for quantitative tissue spectroscopy using the genetic algorithm and inverse Monte Carlo model.

The 15 selected reflectance spectra described from the previous section were used in a wavelength optimization technique that combines our previously developed inverse Monte Carlo model of reflectance [5] with a genetic algorithm (GA) (Global Optimization Toolbox in MATLAB, The MathWorks, Natick, MA). Briefly, the GA uses the principles of natural selection and evolution to produce different solutions for a given problem. For our application, the GA is an appropriate optimization method because it can solve every optimization problem that can be described with chromosome encoding, which is similar to various wavelength combinations. It can also provide multiple solutions for a given problem, which is necessary from a practical system design perspective if not all wavelengths in the solution are available commercially. The algorithm has two major components: (1) the population of individuals (or possible solutions) with its own unique string of “chromosomes” and (2) a fitness function that evaluates the possible solutions. Typically, a population of solutions is randomly generated for a given range of possible solutions. The fitness function is used to evaluate each individual from that population. All of the individuals from the populations are then ranked according to their fitness values. From this existing population, a user-identified proportion is selected to breed a new generation of solutions, and those solutions with fitter values are more likely to be selected. The parent solutions reproduce new offspring solutions by genetic operators such as crossovers or mutations, which essentially results in changes of chromosomes in the offspring and maintains genetic diversity in the subsequent populations. The GA ends when a solution that satisfies the criteria is found, a designated computational time is reached, or a specified generation number is reached. Figure 3 is a general diagram of the steps taken for wavelength optimization, combining an inverse Monte Carlo reflectance model with the GA.

The algorithm begins with the initial constraints of the wavelengths to be used. To extract the 4 parameters of oxy-hemoglobin, deoxy-hemoglobin, β-carotene, and reduced scattering coefficients, at least 5 center wavelengths are needed. The initial population of wavelength sets is created by a random permutation of 50 different wavelength combinations, for 5, 8, and 12 wavelengths in 1, 5, and 10 nm increments from 450–600 nm (151, 31, and 16 possible center wavelengths, respectively). The selection of wavelengths in 1 nm increments represents an ideal situation in system design in which the types of sources available are not limited. The selection of wavelengths in 5 and 10 nm increments represents a more realistic situation, in which the final optimized set of wavelengths will likely be commercially available in the form of bandpass filters. These different wavelength combinations serve as the initial solutions of the iterative GA. The inverse MC model serves as the GA’s fitness function, which is used to evaluate the suitability of each set of wavelengths as a possible solution for extracting <µ_a_>, thus [THb] and [βc], and <µ_s_’> from the training set.

In the 1st generation of a given GA process with its constraint of total wavelengths and the selection increment, the output of the fitness function is 50 sets of extracted breast tissue properties, [THb], [βc], and average <µ_s_’>, for 15 samples using each of the 50 reduced wavelength solution sets. The fitness value was the RMS error between the extracted tissue parameters ([THb], [βc], and average <µ_s_’>) using the reduced wavelength set and using the full 450–600 nm spectrum. Fifty individual wavelength sets were ranked by increasing fitness values (sum of extracted errors) for the 15-sample training set. From these 50, the top 15 wavelength sets with the lowest sum of extracted errors are duplicated to create a new generation of solutions. These same 15 wavelength sets were also used to generate 35 new wavelength sets by means of single-point crossovers or wavelength mutations. In a crossover operation, a random wavelength serves as the point where two wavelength sets break and join. In a mutation operation, a new wavelength is randomly generated from a Gaussian distribution and replaces a wavelength of the parent wavelength set, creating a new wavelength set. The selection of the parent wavelength sets from the previous generation to crossover or to mutate and pass on to the next generation is based on the simulation of a roulette wheel, in which the area of the wheel corresponding to a parent is inversely proportional to the parent’s fitness value, or sum of extracted errors. In other words, the lower the wavelength set’s extracted errors, the higher the probability of that wavelength set is selected, crossed over or mutated, and passed down to be part of the next 50 solutions to be evaluated. Because higher crossover fractions result in less diversity in the subsequent generations and we also found no significant differences in computational time or solutions for various crossover fractions ranging from 0–40%, in this particular study, the crossover fraction is set at 20%, which means 7 of the 35 solutions are the result of a crossover while the remaining 28 are the result of mutations. In the cases of any resulting offspring from a crossover operation having duplicate wavelengths, one of the duplicate wavelengths is discarded, and a new wavelength is randomly generated and inserted in the wavelength set. This process iterates until the minimum fitness value of the generation is unchanged for 10 generations or after 50 generations. All of the GA processes tested in this study converged to an optimum solution given their respective constraints prior to reaching 50 generations. A single optimization requires approximately 21–28 hours, depending on the initial constraints tested, such as the number of wavelengths implemented and the selection increment. The highest ranked 3 solutions from the final generation produced by each GA process were further evaluated using previously described clinical data, independent from the 15 spectra used in the selection process.

#### 1.3 Selection of optimal bandpass

In addition to selecting the most appropriate total number and the center wavelengths of the source, it is also important to understand the effect of increasing full-width half-maximum (FWHM) on the accuracy of the extraction of optical properties. While laser diodes can have very small FWHM, it may not be possible to obtain sources at every wavelength in the optimized solutions. On the other hand, bandpass filters are commercially available at every 10 nm center wavelength in the UV-NIR spectrum, but may come at a cost of 10 nm FWHM around the center wavelength. Light emitting diodes (LEDs) often have even larger FWHM, commonly ranging from 20–50 nm.

Forward Monte Carlo simulations were conducted to study the effect of increasing bandpass. Using the wavelength-dependent optical properties of the 15 clinically measured spectra chosen for the training set described in Section 2.1, diffuse reflectance spectra were generated. The wavelength-dependent absorption coefficients, µ_a_, were determined using the molar extinction coefficients for oxy- and deoxy-hemoglobin, as well as β-carotene. The reduced scattering coefficients, µ_s_’, at each wavelength were calculated using Prahl’s Mie scattering program [18]. The simulations were scaled for the probe geometry used in the clinical measurements [5]. Each of the 15 simulated spectra were convolved with Gaussian distributions of 1, 5, 10, 20, 30, 40, and 50 nm, resulting in a total of 105 spectra of varying FWHM.

The inverse Monte Carlo model was used to extract [THb], [βc], and average <µ_s_’> values from the 15 reflectance spectra with various FWHMs. The extractions were repeated for the top 3 solutions from the wavelength optimizations for 5, 8, and 12 total center wavelengths. The extracted parameters from the reduced wavelengths set with added FWHM were compared to those of the full, simulated spectra without added FWHM. Because these simulations did not include system and measurement artifacts that may exist in measured clinical data, a 10% error was set as the threshold for determining an acceptable FWHM in the analysis.

### 2. Wavelength Selection Validation

#### 2.1 Independent pathology-confirmed tissue data

The results from the wavelength optimization were tested against an existing breast tissue data set, independent of the 15 spectra used for the training set described previously. The inverse Monte Carlo model was used to extract [THb], [βc], and <µ_s_’> from each of the 382 pathological confirmed sites (320 adipose, 24 fibroglandular, 38 malignant) obtained from breast tumor margins. The tissue extractions were performed for the full spectrum of 450–600 nm in 2.5 nm increments for a total of 61 wavelengths, and for the top 3 optimized solutions for each of the test cases: 5, 8, and 12 total wavelengths in 1, 5, and 10 nm increments. To show the differences between optimization and non-optimization, tissue extractions were also made using the semi-evenly spaced wavelengths empirically chosen from 400–600 nm used in a previously reported system [19]. Using the full spectrum [THb], [βc], and <µ_s_’> extractions as the gold standard, errors in the extracted parameters resulting from the reduced wavelength sets were calculated. The Bland-Altman method was used to assess the agreement between the extractions using the full spectrum and the extractions using the optimized and non-optimized spectra.

#### 2.2 Multi-absorber liquid phantom study

A set of 20 phantoms was used to further assess the results from the wavelength optimization. The liquid tissue-simulating phantoms were prepared by mixing polystyrene microspheres (07310, Polysciences, Inc) as the scatterer with water soluble hemoglobin (H0267, Sigma Co.) and crocin (17304, Fluka) as the absorbers. Hemoglobin and crocin were used as the absorbers since they have been used to simulate blood and β-carotene in breast tissue [20]. Based on the optical properties of β-carotene found in previous studies, the appropriate crocin level was added by matching the mean µ_a_ of these two absorbers with similar spectral features [2]. The 2 scattering levels represent the means over 450–600 nm of representative malignant (µ_s_’ = 9 cm^−1^) and normal (µ_s_’ = 12 cm^−1^) breast tissue. The optical properties of the phantoms are shown in Table 2.

**Table 2:** Average µ_a_ (450–600 nm) of liquid phantoms containing hemoglobin, crocin, and polystyrene microspheres.

Absorber Level 1 Avg µ_a_ (cm^−1^)			Absorber Level 2 Avg µ_a_ (cm^−1^)		
Total	Hb	Cr	Total	Hb	Cr
0.51	0.51	0.00	0.91	0.91	0.00
0.99	0.51	0.48	1.72	0.90	0.82
1.23	0.51	0.72	2.12	0.89	1.23
1.47	0.51	0.96	2.53	0.89	1.64
1.70	0.50	1.20	2.93	0.88	2.05

a Each absorber level was tested for 2 scattering levels (avg µ_s_’ = 9 cm^−1^ and avg µ_s_’ = 12 cm^−1^) for a total of 20 phantoms.

The phantom optical measurements were obtained with a previously reported system with slight modifications [1], [9] The system consists of a 450W Xenon Arc lamp and a scanning monochromator (Gemini 180, JY Horiba) coupled to a 600 µm optical fiber as the source. The spectral bandpass of the illumination was fixed at 7 nm. A custom annular silicon photodiode with 2.5 mm outer diameter and 0.75 mm inner diameter was used for detection [21]. The optical fiber was fitted through and epoxied in the detector aperture to illuminate the phantoms, and the detector was connected to a photodiode amplifier (PDA-850, Terahertz Technologies, Inc.) for reflectance measurements. Diffuse reflectance measurements were taken at the discrete wavelength solutions as well as at the evenly spaced wavelengths from 400–600 nm in order to compare the optimized solution to one which samples wavelengths at regularly spaced intervals over the visible spectral range as previously described [8]. The inverse Monte Carlo model was used to extract optical properties from each phantom and root mean square (RMS) errors were compared for both sets of wavelengths.

## Results

### 1. Eight Wavelengths can be Used to Accurately Extract [THb], [βc], and <µ_s_’>

Table 3 enumerates the top 3 solutions for each of the constraints in the optimization, including wavelength range, increment, and total number of wavelengths. For the optimized solutions chosen from 450–600 nm in 1 nm increments, the average errors of extracted THb, βc, and µ_s_’ from the 15 tissue reflectance spectra increases from 11.7% to 12.2% to 18.1% as the total number of wavelengths used decreases from 12 to 8 to 5. When selecting in 5 nm and 10 nm increments, the errors increase from 11.9%–18.5% and 12.0%–19.0% as the wavelengths decrease from 12 to 5, respectively. This trend is not unexpected because as the total number of wavelengths as well as available center wavelength choices decrease, the possibility of capturing the spectral features of the absorbers and scatterers in tissue also decreases, thus increasing the extracted errors.

**Table 3:** Top solutions for each optimization with varied increments and total number of wavelengths.

λ range	# of λs	Optimized Wavelengths	Error
**450∶1∶600**	**12**	451 460 474 483 487 502 511 560 579 584585 596	11.7%
		451 460 474 483 502 509 511 560 573 584585 596	11.8%
		466 479 491 500 516 527 532 560 566 574590 597	12.0%
	**8**	474 481 498 509 555 573 593 596	12.2%
		489 492 503 522 537 558 583 592	12.3%
		485 492 503 510 537 544 560 593	12.5%
	**5**	485 496 512 547 589	18.1%
		478 499 513 582 596	19.5%
		482 496 527 576 597	19.7%
**450∶5∶600**	**12**	460 470 485 490 505 525 530 535 550 570575 600	11.9%
		460 470 475 480 485 500 515 525 530 555585 595	12.2%
		455 465 470 490 505 510 515 530 550 560590 595	12.4%
	**8**	470 485 495 500 510 550 580 600	12.4%
		460 485 500 510 555 560 580 600	12.7%
		450 480 500 505 545 555 585 600	13.2%
	**5**	485 495 510 540 590	18.5%
		490 520 525 570 595	18.7%
		485 495 510 545 595	19.9%
**450∶10∶600**	**12**	450 470 480 490 500 510 520 530 540 560580 600	12.0%
		460 470 480 490 510 530 540 550 560 570580 600	12.1%
		450 460 490 500 510 530 540 550 560 570580 600	12.3%
	**8**	460 490 510 520 540 550 580 600	12.4%
		470 480 490 500 510 560 580 600	12.6%
		480 500 510 530 550 560 570 600	13.3%
	**5**	480 490 520 540 590	19.0%
		480 490 520 550 600	19.9%
		450 490 520 530 590	20.3%

The error from the optimization is the minimized average errors of the extracted parameters from the 15 representative reflectance spectra chosen from the breast tissue data set.

**Figure 4:**
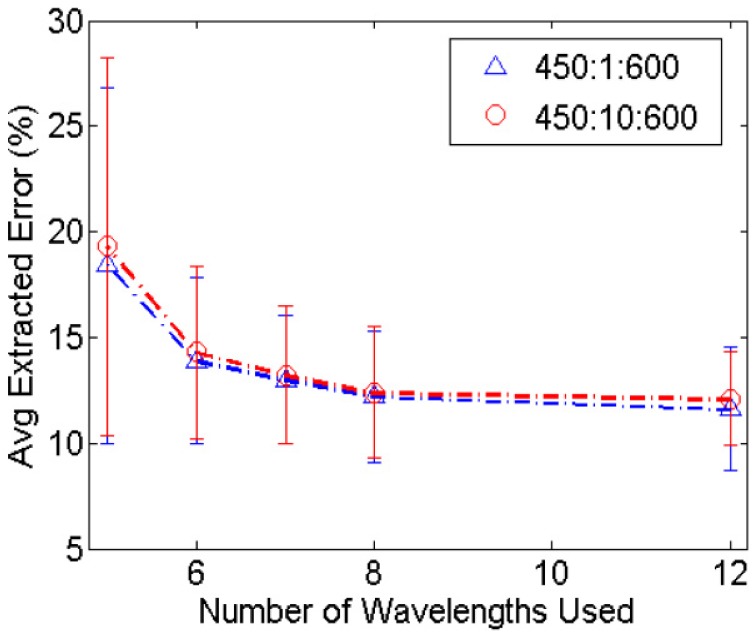
Average of extracted errors for tissue parameters with increasing number of wavelengths. Average extracted % error of [THb], [βc], and <µ_s_’> for 5, 6, 7, 8, and 12 total wavelengths selected from 450–600 nm in 1 and 10 nm increments.

Figure 4 puts into perspective the optimal number of illumination wavelengths required for the design of a breast spectral imaging system. At 5 wavelengths, the average extracted percent error of [THb], [βc], and <µ_s_’> from the 15 representative breast tissue reflectance spectra was close to 20%. The increase to 6, 7, and 8 wavelengths improved the extraction errors to 14%, 13%, and 12%, respectively. There are diminishing returns in improving extraction errors by adding more wavelengths past 8. The graph shows that for our particular application for breast tumor margin assessment, the appropriate number of wavelengths to use is 8.

### 2. Spectral Bandpass Affects Extraction Accuracy

**Figure 5:**
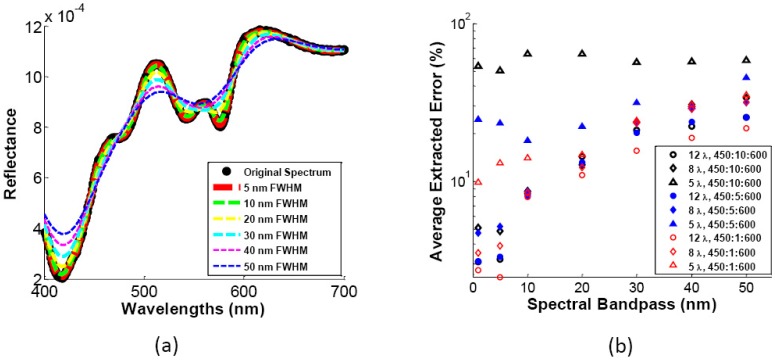
Effect of increasing spectral bandpass. (a) Simulation of the effect increasing spectral bandpass on a diffuse reflectance spectrum representing 10 µM [THb], 5.5 µM [βc], and 3.11 avg <µ_s_’>. (b) Average extracted errors of [THb], [βc], avg <µ_s_’> with increasing spectral bandpass.

Because the system used to obtain the existing breast tumor margin data had a spectral bandpass of 3.9 nm, it is challenging to evaluate the effect of changes in bandpass and to optimize both the wavelengths and bandpass of a system. The forward MC model was used to simulate the same 15 reflectance spectra used in the wavelength optimization. The original spectra were degraded to simulate increases in spectral bandpass of 5, 10, 20, 30, 40, and 50 nm. Representative reflectance spectra (10 µM [THb], 5.5 µM [βc], and 3.11 avg <µ_s_’>) with these changes in spectral bandpass are shown in Figure 5(a). The extracted errors from each case are shown in Figure 5(b). The results in Figure 5(b) show that to extract the breast tissue properties with good accuracy, the wavelengths must have <10 nm FWHM, and 8 or more wavelengths have to be implemented in the system design.

Parameter extraction accuracies are affected by not only the number of wavelengths and the center wavelengths used, but also by the spectral bandpass of the wavelengths. It has been shown through existing clinical data that using 5 wavelengths is likely inadequate for accurate extractions of breast properties. The simulations on the effect of widening spectral bandpass also show that the errors with 5 wavelengths are nearly double those of 8. Similar to previously measured data, the increase from 8 to 12 wavelengths did not seem to have a significant impact on improving the extraction accuracy. Because commercially available LEDs have a much larger bandpass than the 10 nm identified here, more work is required to realize a compact, energy-saving spectral imaging system. Additional filters can be used to narrow the bandpass at each wavelength, or each of the LED spectra can be accounted for with the Monte Carlo reflectance model. Briefly, the shape of each LED spectrum can be added to the MC forward model, which computes a lookup table of “LED-modified” reflectance spectra for a wide range of optical properties. The inverse MC model can then be used to extract optical properties from samples measured with the system with the specified LEDs as sources.

### 3. Optical Contrast in Breast Tissue is Retained with Optimized Wavelength Choices

The top 3 optimized solutions with 5, 8, and 12 total wavelengths selected from 450–600 nm in 1, 5, and 10 nm increments were tested in an independent partial mastectomy tissue data set. Although the initial 15 reflectance spectra selected in the training set spanned the 10^th^ to 90^th^ percentiles of [THb], [βc], and <µ_s_’>, in this large data set the histological diagnoses of the tissues corresponding to these spectra were not known. Therefore, a subset of measurements for which diagnosis was histopathologically confirmed was used to independently test the optimized wavelengths, and were split into 3 tissue types: adipose, fibroglandular, and malignant.

Table 4 and Table 5 provide a summary of all errors extracted for the top 3 optimized solutions for each tissue type and for 5, 8, and 12 wavelengths in 1, 5, and 10 nm increments. A positive percent error value indicates an over-extraction by the reduced-wavelength solutions; a negative value indicates an under-estimation of the extracted parameters. When the number of wavelengths is increased from 8 to 12, the sum of absolute values of the extracted errors for the 3 parameters for any given set of solutions did not improve drastically, which was expected based on the findings shown in Figure 4. When the total number of wavelengths used is decreased from 8 to 5, however, the extracted errors are increased. For solutions selected in 1 nm increments, the extracted [THb] from normal adipose, normal fibroglandular, and malignant tissues using 8 wavelengths differed by 8.5%, −2.6%, and 4.1% from the full 61-wavelength set, respectively. When the wavelengths were reduced to 5, the errors increased to 22.2%, 23.3%, and 21.0% for the 3 tissue types. Similarly, with 8 wavelengths, the extracted [βc] errors for the adipose, fibroglandular, and malignant tissues were −3.0%, −7.0%, and 4.4%. With just 5 wavelengths, the errors increased to −20.8%, 10.9%, and 13.6%. The extracted reduced scattering coefficient errors were also more than doubled (6.6–8.5% to 16.8–22.8%) when total wavelengths decreased from 8 to 5. Because the motivation for this work is to provide a method of optimizing wavelength choices for a compact, cost-effective, and fast spectral imaging device that also has a simplistic design, the solutions with 12 total wavelengths were eliminated and only 8 total wavelengths are used for subsequent system design to be described in a future work.

**Table 4:** Summary of average extracted errors of parameters for various tissue types for the top 3 optimized solutions.

Constraints		[THb]			[βc]			<µ_s_’>		
# of λ, increment	Tissue	Set1	Set2	Set3	Set1	Set2	Set3	Set1	Set2	Set3
12λ: 450∶1∶600	A	3.1	2.4	4.5	−3.9	−5.5	−10.8	9.3	3.0	7.8
	FG	3.9	3.4	5.6	−13.9	−15.9	−15.7	4.2	3.9	11.8
	M	3.0	3.9	−4.8	2.1	−2.5	−11.9	8.9	2.1	5.3
12λ: 450∶5∶600	A	3.8	2.0	5.6	−7.9	−4.6	−2.4	6.4	11.7	2.7
	FG	2.0	8.8	12.2	−7.6	−10.1	−11.4	11.2	4.9	10.0
	M	−2.8	6.0	5.9	−2.7	−5.2	−7.0	2.7	6.2	7.7
12λ: 450∶10∶600	A	4.0	−2.2	6.0	−2.8	−8.7	−11.5	1.4	3.8	9.3
	FG	7.7	9.7	10.4	−9.6	−17.3	−14.9	3.2	10.8	15.7
	M	6.6	−6.5	4.7	−4.4	−8.5	−9.5	2.0	−4.0	7.7
8λ: 450∶1∶600	A	8.5	5.5	11.0	−3.0	−1.4	−5.7	6.6	8.8	7.9
	FG	−2.6	10.4	8.7	−7.0	−18.9	−12.0	6.6	13.5	11.1
	M	4.1	−8.2	11.9	4.4	−3.0	3.2	8.5	5.0	7.7
8λ: 450∶5∶600	A	7.1	7.0	8.2	−2.9	−4.7	−6.1	8.6	9.0	8.9
	FG	5.9	8.5	11.9	−6.8	−18.2	−10.0	13.3	14.6	15.5
	M	5.3	7.7	8.2	2.9	−7.7	2.1	7.1	8.7	8.7
8λ: 450∶10∶600	A	11.7	6.7	13.9	−12.2	−4.8	−13.2	4.8	2.2	9.1
	FG	13.9	7.2	7.1	−15.4	−8.8	−10.0	9.0	4.5	14.8
	M	−11.8	7.5	12.9	−19.2	−2.9	−9.4	1.1	2.8	8.1
5λ: 450∶1∶600	A	22.2	23.3	21.0	−20.8	−10.2	10.1	17.2	16.6	15.6
	FG	23.3	28.4	27.0	10.9	−18.1	12.5	22.8	23.5	23.2
	M	21.0	25.3	25.9	13.6	11.8	13.8	16.8	18.2	18.3
5λ: 450∶5∶600	A	22.3	26.4	23.4	23.7	−23.2	24.0	18.4	18.7	18.2
	FG	27.1	29.3	20.9	28.1	−20.6	28.0	25.1	23.5	24.4
	M	23.4	26.6	22.8	18.8	−21.2	18.7	19.6	25.4	18.3
5λ: 450∶10∶600	A	16.0	21.4	24.4	26.9	28.8	25.1	24.7	24.9	21.9
	FG	13.3	21.8	17.9	22.5	23.8	28.1	29.6	29.8	29.0
	M	19.7	15.6	−20.3	25.1	23.4	−21.5	20.9	22.0	−21.1
ES8λ: 400–600	A	22.5			14.0			−15.5		
	FG	23.6			64.4			−18.3		
	M	17.3			15.7			−26.3		

Positive values indicate an over-estimation of the extracted parameters while negative values indicate an under-estimation of the parameters. [THb]: total hemoglobin; [βc]: β-carotene; <µ_s_’>: reduced scattering coefficient; A: adipose tissue; FG: fibroglandular; M: malignant tissue; ES8λ: semi-evenly spaced 8 wavelengths (400, 420, 440, 470, 500, 530, 570, 600 nm).

**Table 5:** Summary of average extracted errors of the ratio of [THb]/<µ_s_’> and [βc]/<µ_s_’> for various tissue types for the top 3 optimized solutions.

Constraints		[THb]/<µ_s_’>			[βc]/<µ_s_’>		
# of λ, increment	Tissue	Set1	Set2	Set3	Set1	Set2	Set3
12λ: 450∶1∶600	A	−2.9	−4.5	−8.4	−15.4	−6.6	−10.8
	FG	−7.0	−1.5	−13.3	−13.1	−10.9	−12.7
	M	−3.7	−4.2	−8.3	−7.9	−2.1	−8.7
12λ: 450∶5∶600	A	−6.1	0.3	0.7	−5.6	−5.7	−9.8
	FG	−9.8	−7.7	−4.9	−12.8	−15.9	−13.1
	M	−6.4	−0.5	−2.2	−6.3	−11.7	−8.9
12λ: 450∶10∶600	A	−3.0	−6.5	−4.1	−6.1	−8.8	−13.7
	FG	−6.0	−8.4	−6.9	−12.2	−12.8	−16.8
	M	−3.4	−3.9	−4.0	−5.6	−9.8	−9.2
8λ: 450∶1∶600	A	−4.8	−6.0	−7.3	−11.3	−12.7	−15.1
	FG	−1.4	−1.5	−1.2	−10.1	−18.6	−16.4
	M	−2.5	−6.1	−6.9	−8.5	−9.6	−5.1
8λ: 450∶5∶600	A	−1.8	−2.6	−5.9	−3.4	−16.9	−17.3
	FG	−5.3	−7.4	−4.7	−14.1	−12.0	−9.9
	M	−2.5	−1.7	−1.3	−5.2	−9.0	−7.6
8λ: 450∶10∶600	A	−8.6	4.2	−6.0	8.3	7.6	15.0
	FG	−5.8	2.2	−9.4	19.0	14.4	19.9
	M	−8.5	4.4	−6.3	10.0	4.3	9.6
5λ: 450∶1∶600	A	17.4	18.4	16.7	−21.6	−32.3	−18.4
	FG	13.1	16.3	12.7	−28.7	−30.4	−24.0
	M	15.3	21.0	19.8	−23.5	−19.8	−14.7
5λ: 450∶5∶600	A	16.1	−20.4	17.3	−28.0	−21.9	−27.6
	FG	14.6	−18.3	14.4	−23.8	−32.8	−22.4
	M	15.9	−23.7	16.1	−23.3	−22.8	20.2
5λ: 450∶10∶600	A	−17.6	−16.7	−17.7	23.1	28.8	−26.9
	FG	−17.4	−17.2	−16.7	−23.5	24.3	−20.8
	M	−22.0	25.8	−18.0	−21.2	24.8	−20.9
ES8λ: 400–600	A	44.0			35.3		
	FG	26.6			102.1		
	M	70.1			75.5		

Positive values indicate an over-estimation of the extracted parameters while negative values indicate an under-estimation of the parameters. [THb]: total hemoglobin; [βc]: β-carotene; <µ_s_’>: reduced scattering coefficient; A: adipose tissue; FG: fibroglandular; M: malignant tissue; ES8λ: semi-evenly spaced 8 wavelengths (400, 420, 440, 470, 500, 530, 570, 600 nm).

Although the top optimized solution for each wavelength selection constraint had the lowest average errors of [THb], [βc], and <µ_s_’> extracted from the 15 representative reflectance spectra in the training set, the best choice of wavelengths from the independent breast data set validation is not necessarily the same as the best solution from the training set. This is possibly due to the relatively small size of the training set. However, the differences in errors between the top 3 optimized solutions are also small, which indicates that the solutions have been minimized. The best wavelength set for extracting optical parameters with the lowest errors in the histopathology confirmed data set is solution #1: 474, 481, 498, 509, 555, 573, 593, 596 nm for the wavelengths selected in 1 nm increments. The best wavelength set selected from 5 nm increments is also solution #1: 470, 480, 495, 500, 510, 550, 580, 600 nm. However, the best wavelength set selected from 10 nm increments is solution #2: 470, 480, 490, 500, 510, 560, 580, 600 nm. The wavelengths selected from 1 nm increments from 450–600 do have striking similarities with those selected from both the 5 nm and 10 nm increments. From a practical system design standpoint, this is a good finding because of the abundant availability of sources in 10 nm increments, such as those of LEDs. On the other hand, the wavelengths selected from 1 nm increments (and some wavelengths in 5 nm increments) in the 450–600 nm range are not all commercially available to date. Since wavelength choices in both the 5 nm and 10 nm increments did not yield significantly different extracted errors, the 8 wavelengths selected in 10 nm increments (solution #2) were chosen for subsequent analyses in this study: 470, 480, 490, 500, 510, 560, 580, and 600 nm, which are all commercially available filters with 10 nm bandpasses. A practical low-cost implementation for these center wavelengths would be to use a white LED together with the respective bandpass filters.

The optimization helped identify wavelength sets that can be used to extract tissue parameters with errors <20%; however, the acceptable extraction errors for [THb], [βc], and <µ_s_’> has to be determined based on the contrast of these optical endpoints in various tissue types. In Table 6, the percent difference of quantifiable optical contrast was calculated between the histologically-confirmed median adipose and malignant tissue samples, as well as the median fibroglandular and the malignant samples. A positive percent difference indicates that the benign (adipose or fibroglandular) tissue samples had greater extracted values than those of the malignant sample. The malignant sample showed decreased [THb] and <µ_s_’> compared to the adipose samples and decreased [THb] and [βc] compared to the fibroglandular sample. Also in the table are the extraction percent changes from the full 450–600 nm spectrum compared to the 8 optimized wavelengths and the 8 evenly spaced wavelengths. A positive percent change indicates an over-estimation of the extracted values by the 8-wavelength reduced spectra compared to the full 450–600 nm spectrum. A negative percent change means that the extracted values are decreased using 8 wavelengths.

**Table 6:** Comparison of the percent difference between median adipose and malignant tissue and fibroglandular and malignant tissue to the percent change of extractions using the optimized wavelengths and evenly spaced wavelengths to the full 450–600 nm spectrum.

Medians	% Difference		Extraction % change from full 450–600 nm spectrum					
	Full spectrum (450–600)		Optimized 8 λs			Semi-evenly spaced 8 λs		
	A vs. M	FG vs. M	A	FG	M	A	FG	M
[THb]	−40.66	−25.16	8.90	10.48	2.63	−15.09	5.64	−1.28
[βc]	9.90	−9.54	−4.54	−8.74	−0.76	−2.74	−35.39	−6.79
<µ_s_’>	−36.89	22.83	1.15	11.72	2.21	18.05	26.88	27.89
[THb]/<µ_s_’>	−2.75	−62.18	1.84	−1.40	0.43	−40.43	−29.04	−40.45
[βc]/<µ_s_’>	13.07	−87.48	−5.75	−23.18	21.99	−25.36	−85.15	−12.12

[THb]: total hemoglobin; [βc]: β-carotene; <µ_s_’>: reduced scattering coefficient; A: adipose tissue; FG: fibroglandular; M: malignant tissue; Positive percent difference indicates that the benign tissues (A and FG) had greater extracted values; negative percent difference means the malignant sites were greater. A positive extraction percent change indicates an over-estimation of the extracted parameters while a negative percent change indicates an under-estimation of the parameters.

The percent change using the optimized wavelengths is smaller than the percent difference for all optical parameters so optical contrast should be preserved with these reduced wavelengths. On the flip side, the percent change using the evenly spaced wavelengths without any optimization is sometimes greater than the percent difference for the optical parameters, such as for [βc] and [βc]/<µ_s_’>. This means that the optical contrast to differentiate benign from malignant samples may be washed out if the un-optimized wavelengths are used. By examining the percent differences between optical parameters of benign and malignant samples and the mean extracted errors from Table 4 and Table 5, it was also further established that none of the top wavelength sets with only 5 wavelengths can be used for our clinical application because most of the extracted errors are greater than percent differences of the optical parameters for the various breast tissue types tested.

**Figure 6:**
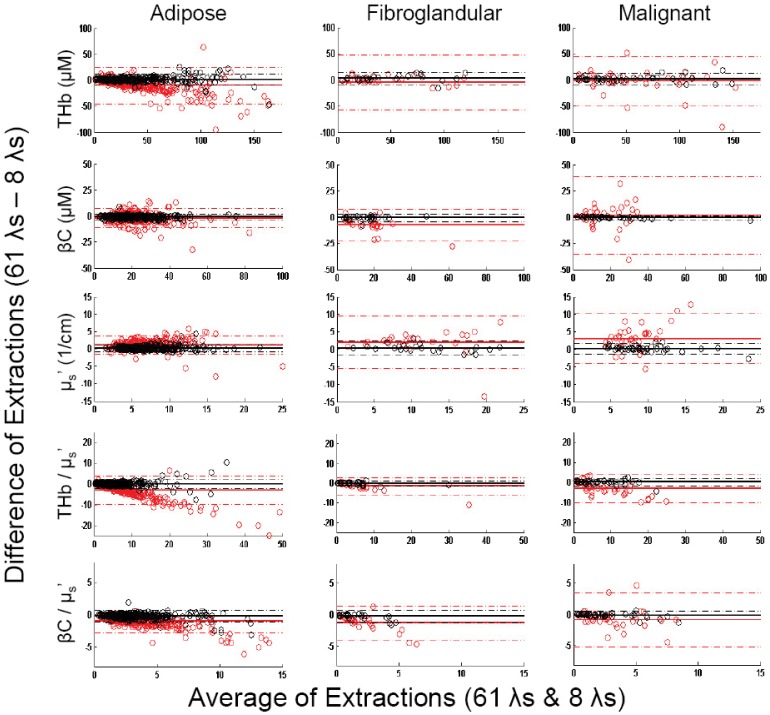
Bland-Altman plots of MC extractions using various wavelength combinations. Bland-Altman plots assessing the agreement of MC extractions of [THb], [βc], <µ_s_’>, [THb]/<µ_s_’>, and [βc]/<µ_s_’> in adipose, fibroglandular, and malignant tissue types using the full spectrum versus the optimized reduced wavelength spectrum with 8 wavelengths (470, 480, 490, 500, 510, 560, 580, 600 nm) shown in black and the regularly spaced intervals (400, 420, 440, 470, 500, 530, 570, 600 nm) shown in red. The solid lines indicate the mean difference (bias) between the extractions; the dashed lines indicate the 95% limits of agreement.

Figure 6 shows the Bland-Altman plots comparing the extractions between the optimum 8-wavelength set and the evenly spaced 8-wavelength set with the full 450–600 nm 61-wavelength set. The various tissue types are shown in columns, and the extracted parameters are shown in rows. The mean difference (or bias between the optimized reduced and full wavelength extractions) and 95% limits of agreement for [THb] are 1.5±10.6, 3.2±11.6, and 2.3±11.3 µM for adipose, fibroglandular, and malignant tissue types, respectively. By comparison, the evenly spaced 8-wavelength extractions do not agree as well for extracting [THb], with the mean difference and 95% limits of agreement at −10.0±35.2, −4.4±51.8, and −2.2±46.3 µM for the three tissue types. Similar trends are observed for the extraction of [βc] and <µ_s_'>. The mean differences between the opimized 8 wavelengths and the 61-wavelength spectrum for the extraction of [βc] are close to 0 with a much smaller range of limits of agreement for various tissue types: −0.7±2.7, −0.7±3.9, and −0.4±1.7 µM. By comparison, the un-optimized evenly spaced wavelengths have a larger difference and wider range: −1.9±9.3, −7.1±14.9, and 1.8±37.1 µM. For scattering, the optimal wavelengths also performed better: 0.2±1.1, 0.3±2.0, and 0.2±1.6 cm^−1^ compared to 1.1±2.5, 2.0±7.5, and 3.1±7.0 cm^−1^. Because previous studies have shown that [βc] and <µ_s_’> are significant parameters that provide optical contrast for breast tumor margin assessment, these results further show the importance of optimizing wavelength choices.

**Figure 7:**
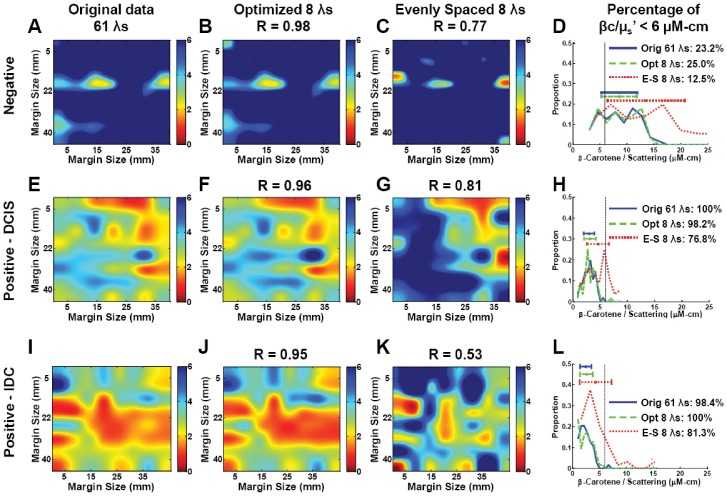
Example spectral images of negative and positive margins obtained with and without optimization. Representative margin maps of [βc]/<µ_s_’> for normal (A–C), ductal carcinoma in situ (E–G), and invasive ductal carcinoma (I–K) using the full 450–600 nm spectrum, the optimized 8 wavelengths, and the un-optimized evenly spaced 8 wavelengths. Corresponding correlation coefficients for the 61-wavelength spectra and the reduced 8-wavelength spectra are shown. Distribution of the extracted βc/µ_s_’ are shown in (D), (H), and (L) for each case, along with the threshold values used in the predictive model to separate positive from negative margins.

Figure 7 contains representative breast tumor margin images of extracted [βc]/<µ_s_’> for a negative (normal) and two positive breast resection margins: ductal carcinoma *in situ* (DCIS) and invasive ductal carcinoma (IDC). The margin images shown in (a), (e), and (i) were obtained using the full 450–600 nm spectrum. The images shown in (b), (f), and (j) were extracted using the optimized solution for 8 wavelengths: 470, 480, 490, 500, 510, 560, 580, and 600 nm. The images shown in (c), (g), and (k) were extracted using the evenly spaced 8 wavelengths used in a previous system: 400, 420, 440, 470, 500, 530, 570, and 600 nm. The correlation coefficients for the images extracted with optimized 8 wavelengths (as compared to the images extracted using the full 61-wavelength set) were 0.98, 0.96, and 0.95 for the normal, DCIS, and IDC margins, respectively. The correlation coefficients for the evenly spaced 8 wavelengths were 0.77, 0.81, and 0.53. Histograms are shown in (d), (h), and (l) to compare the extracted [βc]/<µ_s_’> using 61 wavelengths versus just 8 wavelengths with and without optimization. Wilke et al. reported using a threshold of 6 for the [βc]/<µ_s_’> ratio for classifying negative and positive margins [7]. If 98% of the pixels that make up the margins have a ratio <6, the margin is then classified as positive. The histogram shows that with the optimized 8 wavelengths, the contrast in breast margins is preserved. Without the optimization, some contrast is lost. These margin maps can potentially help surgeons identify suspicious “hot spots,” where cancer cells may be present at the surface of the excised specimen.

**Figure 8:**
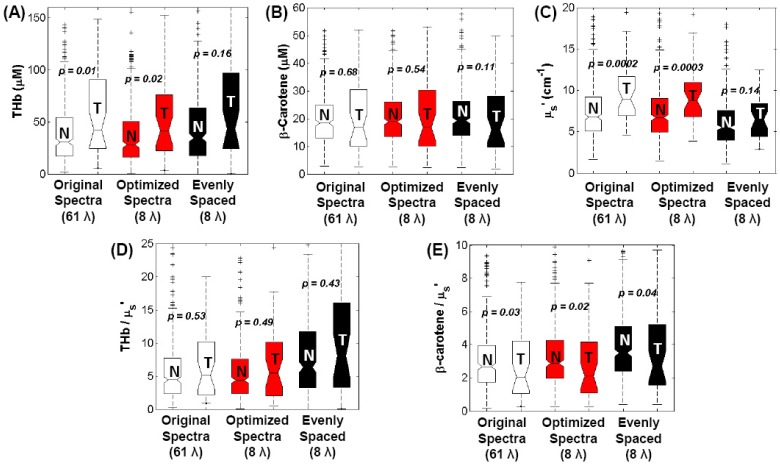
Comparison of Monte Carlo extractions of normal and cancerous tissue parameters. Comparison of the MC extractions of [THb], [βc], <µ_s_’>, [THb]/<µ_s_’>, and [βc]/<µ_s_’> in adipose, fibroglandular, and malignant tissue types using full spectrum versus the optimized reduced wavelength spectrum and evenly spaced spectrum with 8 wavelengths. Sample sizes are Normal (N) = 344, and Tumor (T) = 38.

Wilcoxon Rank Sum tests were performed to compare the Monte Carlo extracted optical properties using the full 61 and reduced wavelengths, both the optimized and evenly spaced 8. The boxplots of the comparisons are shown in Figure 8. The histologically normal samples were comprised of 320 adipose and 24 fibroglandular samples (total N = 344) compared to the 38 malignant samples. The extractions of [THb], [βc], and <µ_s_’> using the optimized 8 and 61 wavelengths were not significantly different for all tissue types. The findings from an observational study on the effects of tissue heterogeneity reported by Kennedy et al were also duplicated [17]. [THb] and <µ_s_’> were both significantly increased in the malignant samples compared to the normal samples. Using the evenly spaced 8 wavelengths that were selected empirically for a previous system, the extracted <µ_s_’> is most notably underestimated for malignant samples while the [THb] and the ratio [βc]/<µ_s_’> are overestimated. These are consistent with the Bland-Altman plots shown in Figure 6. Without wavelength optimization, the contrast between benign and malignant samples for [THb] and <µ_s_’> is not retained as wavelength numbers are reduced to the 8 evenly spaced wavelengths. Although these results show that a reduced wavelength set can be used in place of the full wavelength spectrum to obtain optical contrast in previously acquired breast tissue data, which have disproportionally large number of adipose normal tissue, the main goal of this study is not to show the predictive power for separating normal from tumor, but rather it is to find a reduced number of wavelengths that can be used to extract reasonably similar tissue parameters compared to the full spectrum. With the ability to extract similar tissue parameters from previous clinical studies reported by Wilke et al. and Kennedy et al., we expect to have similar success in classification in future studies with a new compact device with the optimized wavelengths implemented.

### 4. Wavelength Optimization Improves Extraction Accuracy in Phantoms

**Figure 9:**
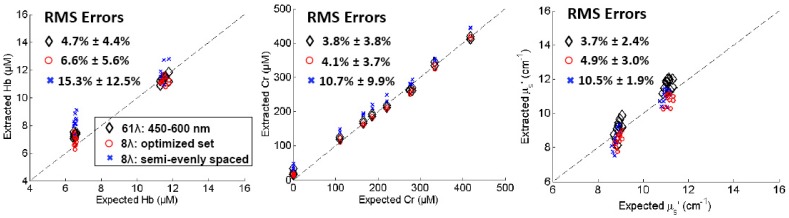
Multi-absorber phantom optical properties extracted with and without optimization. Comparison of extraction accuracy for [Hb], [Cr], and <µ_s_’> using the full 450–600 nm spectrum, the optimized wavelength solution, and the evenly spaced wavelengths selected empirically for a previously reported system.

Figure 9 compares the extraction accuracy in the multi-absorber liquid phantom study using the full 450–600 nm range, the optimized wavelengths, and the evenly spaced wavelengths that were chosen empirically for a previously reported system [22]. The RMS errors for the extraction of [Hb], [Cr], and <µ_s_’> using the 61 wavelengths in the 450–600 nm range were 4.7±4.4%, 3.8±3.8%, and 3.7±2.4%, respectively. However, using the 8 evenly spaced wavelengths without any optimization, the RMS errors are 15.3±12.5% for [Hb], 10.7±9.9% for [Cr], and 10.5±1.9% for <µ_s_’>. With the optimized 8 wavelengths, the RMS errors of extracted [Hb], [Cr], and <µ_s_’> were decreased to 6.6±5.6%, 4.1±3.7%, and 4.9±3.0%, respectively. These errors are not significantly different from the errors from the full 450–600 nm spectrum. Referring back to Table 6 for an approximation of acceptable errors, these phantom results show the benefit of wavelength optimization for extracting hemoglobin and a β-carotene substitute while maintaining optical contrast, which is of utmost importance for our application.

### Conclusions

A method that combines a genetic algorithm and inverse Monte Carlo reflectance model was applied and validated in an independent clinical dataset to systematically select wavelengths and bandwidths in the design of a spectral imaging system for the application of breast tumor margin assessment. The development of this method was motivated by the system design for a compact, cost-effective spectral imaging system, which features a white LED with bandpass filters. We demonstrate that at least 5 wavelengths are required to extract oxy- and deoxy-Hb, βc, and µ_s_’ for this acquisition geometry. We found that the minimum number of wavelengths to retain optical contrast obtained from a full 450–600 nm set is 8 wavelengths. Designing a system with additional wavelengths up to 12 provides minimal improvements in extraction errors at a potentially higher cost of increasing system footprint, data acquisition time, and system design complexity. Additionally, a two-absorber turbid phantom study showed improved quantitative accuracy for optimized wavelength sets. This method may be adapted to the optimization of other quantitative spectroscopic imaging instruments in clinical applications beyond breast tumor margin assessment.

## References

[1] Dhar S, Lo JY, Palmer GM, Brooke MA, Nichols BS, et al (2012) A diffuse reflectance spectral imaging system for tumor margin assessment using custom annular photodiode arrays. Biomedical Optics Express 3: 3211–3222. 23243571 10.1364/BOE.3.003211 PMC3521310

[2] Bydlon TM, Kennedy SA, Richards LM, Brown J, Yu B, et al (2010) Performance metrics of an optical spectral imaging system for intra-operative assessment of breast tumor margins. Optics Express 18: 8058–8076. 20588651 10.1364/OE.18.008058 PMC2939901

[3] Poggi MM, Danforth DN, Sciuto LC, Smith SL, Steinberg SM, et al (2003) Eighteen-year results in the treatment of early breast carcinoma with mastectomy versus breast conservation therapy. Cancer 98: 697–702. 12910512 10.1002/cncr.11580

[4] Clarke M, Collins R, Darby S, Davies C, Elphinstone P, et al (2005) Effects of radiotherapy and of differences in the extent of surgery for early breast cancer on local recurrence and 15-year survival: an overview of the randomised trials. Lancet 366: 2087–2106. 16360786 10.1016/S0140-6736(05)67887-7

[5] Palmer GM, Ramanujam N (2006) Monte Carlo-based inverse model for calculating tissue optical properties. Part I: Theory and validation on synthetic phantoms. Applied optics 45: 1062–1071. 16512550 10.1364/ao.45.001062

[6] Palmer GM, Zhu C, Breslin TM, Xu F, Gilchrist KW, et al (2006) Monte Carlo-based inverse model for calculating tissue optical properties. Part II: Application to breast cancer diagnosis. Applied optics 45: 1072–1078. 16512551 10.1364/ao.45.001072

[7] Wilke LG, Brown JQ, Bydlon TM, Kennedy SA, Richards LM, et al (2009) Rapid noninvasive optical imaging of tissue composition in breast tumor margins. The American Journal of Surgery 198: 566–574. 19800470 10.1016/j.amjsurg.2009.06.018 PMC2764289

[8] Fu HL, Yu B, Lo JY, Palmer GM, Kuech TF, et al (2010) A low-cost, portable, and quantitative spectral imaging system for application to biological tissues. Optics Express 18: 12630–12645. 20588390 10.1364/OE.18.012630

[9] Lo JY, Yu B, Fu HL, Bender JE, Palmer GM, et al (2009) A strategy for quantitative spectral imaging of tissue absorption and scattering using light emitting diodes and photodiodes. Optics Express 17: 1372–1384. 19188966 10.1364/oe.17.001372

[10] Yu B, Lo JY, Kuech TF, Palmer GM, Bender JE, et al (2008) Cost-effective diffuse reflectance spectroscopy device for quantifying tissue absorption and scattering in vivo. Journal of Biomedical Optics 13: 060505. 19123646 10.1117/1.3041500

[11] Mazhar A, Dell S, Cuccia DJ, Gioux S, Durkin AJ, et al (2010) Wavelength optimization for rapid chromophore mapping using spatial frequency domain imaging. Journal of Biomedical Optics 15: 061716–061719. 21198164 10.1117/1.3523373 PMC3031903

[12] Umeyama S, Yamada T (2009) New cross-talk measure of near-infrared spectroscopy and its application to wavelength combination optimization. Journal of Biomedical Optics 14: 034017–034018. 19566310 10.1117/1.3147402

[13] Ferreira DS, Mirkovic J, Wolffenbuttel RF, Correia JH, Feld MS, et al (2011) Narrow-band pass filter array for integrated opto-electronic spectroscopy detectors to assess esophageal tissue. Biomed Opt Express 2: 1703–1716. 21698030 10.1364/BOE.2.001703 PMC3114235

[14] Phelps JE, Vishwanath K, Chang VTC, Ramanujam N (2010) Rapid ratiometric determination of hemoglobin concentration using UV-VIS diffuse reflectance at isosbestic wavelengths. Opt Express 18: 18779–18792. 20940771 10.1364/OE.18.018779 PMC3093134

[15] Bigio IJ, Bown SG (2004) Spectroscopic sensing of cancer and cancer therapy: current status of translational research. Cancer Biol Ther 3: 259–267. 15107613 10.4161/cbt.3.3.694

[16] Brown J, Wilke LG, Geradts J, Kennedy SA, Palmer GM, et al (2009) Quantitative optical spectroscopy: a robust tool for direct measurement of breast cancer vascular oxygenation and total hemoglobin content in vivo. Cancer research 69: 2919. 19293184 10.1158/0008-5472.CAN-08-3370 PMC2677720

[17] Kennedy S, Geradts J, Bydlon T, Brown JQ, Gallagher J, et al (2010) Optical breast cancer margin assessment: an observational study of the effects of tissue heterogeneity on optical contrast. Breast Cancer Research 12: R91. 21054873 10.1186/bcr2770 PMC3046432

[18] Prahl S (2005) Mie scattering program. Oregon Medical Laser Center.

[19] Lo JY, Dhar S, Yu B, Brooke MA, Kuech TF, et al (2012) Diffuse reflectance spectral imaging for breast tumor margin assessment. Proceedings of SPIE 8214: 821407.

[20] Bender JE, Vishwanath K, Moore LK, Brown JQ, Chang V, et al (2009) A robust Monte Carlo model for the extraction of biological absorption and scattering in vivo. Biomedical Engineering, IEEE Transactions on 56: 960–968. 10.1109/TBME.2008.2005994 PMC2791541 19423425

[21] Dhar S, Lo JY, Yu B, Brooke MA, Ramanujam N, et al.. (2011) Custom annular photodetector arrays for breast cancer margin assessment using diffuse reflectance spectroscopy. IEEE. 440–443.

[22] Lo JY, Yu B, Kuech TF, Ramanujam N (2011) A compact, cost-effective diffuse reflectance spectroscopic imaging system for quantitative tissue absorption and scattering. Proceedings of SPIE 7890: 78900B.

